# Oral cow’s milk immunotherapy: clinical and serological data in long-term follow up

**DOI:** 10.1186/2045-7022-5-S3-P158

**Published:** 2015-03-30

**Authors:** Paloma Poza Guedes, Ruperto González Pérez, Inmaculada Sánchez Machín, Victor Matheu Delgado

**Affiliations:** 1 Santa Cruz de Tenerife, Canary Islands, Spain

## Rationale

Oral food inmunotherapy is a promising therapeutic aproach in patients with persistent cow's milk allergy (CMA). Although it seems that these protocols show a better outcome in patients with "milder" symptoms (i.e. non anaphylactic reactions) there are controversial results in highly sensitized subjects.

## Methods

We select patients with persistent CMA and severe uncontrolled anaphylactic symptoms despite a correct restrictive diet. We performed a two-day desensitization procedure at the Pediatric Critical Care Unit in our Institution. The second phase was weekly scheduled in the Outpatient clinic to reach a final cumulative dose of 250 ml of undiluted milk. Clinical and serological date were collected every six month for a five-year period.

## Results

Fifteen children (2-16 y.o.) were included. All children reached the final dose of 250 ml of undiluted milk in less than ten weeks. Clinical follow-up every 6 months remained during 5 years to register all adverse reactions and possible factors involved. Serological changes were obtained every six months during the subsequent five years, including specific IgE and IgG4.

## Conclusion

Anaphylactic CMA patients may benefit from rush oral Cow's Milk immunotherapy. Clinical and serological changes have been found both at early and long-term follow-up. Several factors were involved in reactions for temporary loss of tolerance.

